# The “Jack-of-all-Trades” Flagellum From *Salmonella* and *E. coli* Was Horizontally Acquired From an Ancestral β-Proteobacterium

**DOI:** 10.3389/fmicb.2021.643180

**Published:** 2021-03-30

**Authors:** Josie L. Ferreira, Izaak Coleman, Max L. Addison, Tobias Zachs, Bonnie L. Quigley, Kristin Wuichet, Morgan Beeby

**Affiliations:** ^1^Department of Life Sciences, Imperial College London, London, United Kingdom; ^2^Department of Biomedical Informatics, Vanderbilt University Medical Center, Nashville, TN, United States

**Keywords:** bacterial flagella, electron cryotomography, molecular evolution, subtomogram averaging, horizontal gene transfer

## Abstract

The γ-proteobacteria are a group of diverse bacteria including pathogenic *Escherichia, Salmonella, Vibrio*, and *Pseudomonas* species. The majority swim in liquids with polar, sodium-driven flagella and swarm on surfaces with lateral, non-chemotactic flagella. Notable exceptions are the enteric Enterobacteriaceae such as *Salmonella* and *E. coli*. Many of the well-studied Enterobacteriaceae are gut bacteria that both swim and swarm with the same proton-driven peritrichous flagella. How different flagella evolved in closely related lineages, however, has remained unclear. Here, we describe our phylogenetic finding that Enterobacteriaceae flagella are not native polar or lateral γ-proteobacterial flagella but were horizontally acquired from an ancestral β-proteobacterium. Using electron cryo-tomography and subtomogram averaging, we confirmed that Enterobacteriaceae flagellar motors resemble contemporary β-proteobacterial motors and are distinct to the polar and lateral motors of other γ-proteobacteria. Structural comparisons support a model in which γ-proteobacterial motors have specialized, suggesting that acquisition of a β-proteobacterial flagellum may have been beneficial as a general-purpose motor suitable for adjusting to diverse conditions. This acquisition may have played a role in the development of the enteric lifestyle.

## Introduction

Understanding molecular evolution is fundamental to contemporary biology. Compared to evolutionary processes in large eukaryotes, however, relatively little is known about how molecular machines are acquired, adapted, or change function, and how this relates to the environment. One of the most iconic molecular machines is the bacterial flagellar motor, a self-assembling molecular machine that harnesses ion flux for propulsion.

The best studied flagella are the peritrichous (randomly positioned) motors from the Enterobacteriaceae (henceforth, “enterics”) *Salmonella enterica* and *Escherichia coli*, which are used for both aquatic swimming and surface-based swarming. Flagellar rotation is driven by a ring of stator complexes, which incorporate dynamically as a function of load (Reid et al., 2006; Tipping et al., 2013). Ion flux through the stator complexes rotates a cytoplasmic C-ring; torque is transmitted to the extracellular flagellum via a rigid rod and short universal joint. Enterics swim with a biased random walk: when all flagella rotate counterclockwise, the universal joints facilitate bundling of flagella for propulsion. Binding of the response regulator CheY to the C-ring triggers clockwise rotation, disrupting the flagellar bundle and randomly reorienting the cell (Lee et al., 2001). By phosphorylating CheY when swimming down favorable gradients (or up unfavorable gradients), the bacterium reorients more frequently, leading to a random walk that is biased away from detrimental environments and toward favorable environments (Berg and Brown, 1972; Silverman and Simon, 1974).

Non-enteric γ-proteobacteria such as *Vibrio, Shewanella*, *Pseudomonas*, and *Plesiomonas* swim differently from the enterics. Instead of multifunctional peritrichous flagella, non-enteric γ-proteobacteria have high-torque polar motors, usually Na^+^-driven, with high-occupancy stator complexes held in place by large periplasmic disks (Terashima et al., 2006, 2010; Beeby et al., 2016; Zhu et al., 2017); many γ-proteobacteria also have secondary lateral flagella used for surface-based swarming motility, or as “rudders” (Bubendorfer et al., 2014). For chemotaxis, polar motors reorient the cell using a “forward-reverse-flick” motion instead of disrupting the bundle (Xie et al., 2011), while lateral motors are non-chemotactic.

Although previous studies have investigated other incongruencies between flagellar systems and the bacteria they appear in Liu and Ochman (2007); Poggio et al. (2007), the basis of the aforementioned long-known differences between model enteric flagella and flagella in other, closely-related γ-proteobacteria, is unclear. As well as differences in function, the peritrichous, polar, and lateral flagella have distinct wave amplitudes and frequencies (Fujii et al., 2008), suggesting that they come from different families, differences that correlate with habitat: many of the well-studied enterics are gut-dwelling pathogens and commensals. Here, we describe our investigations of the relationship between the different flagellar systems. Phylogenetic and structural results reveal that the enterics acquired a β-proteobacterial flagellar motor by horizontal transfer. This transfer may have provided contemporary enteric bacteria with a general-purpose motor better able to adjust its behavior to a wide range of environmental conditions than the more specialized motors native to the γ-proteobacteria.

## Results and Discussion

**FIGURE 1:**
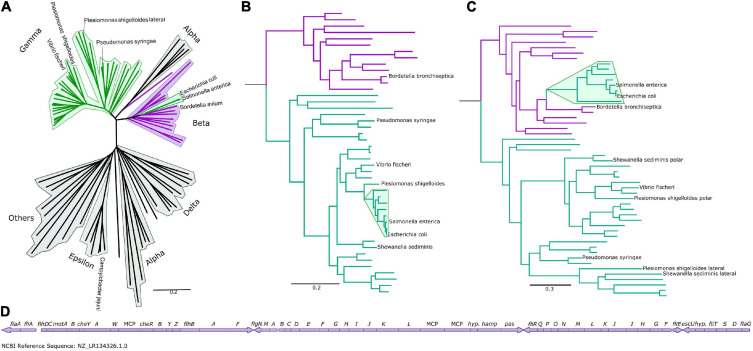
The Enterobacteriaceae have β-like motors. **(A)** An unrooted global flagellar phylogeny. γ-proteobacteria are highlighted in green: the enteric Enterobacteriaceae γ-proteobacteria (*Salmonella enterica* and *Escherichia coli)* are not clustered with the other γ-proteobacteria, but are clustered within the β-proteobacteria (purple). Fully annotated version of this tree is presented in Supplementary Figure S1. **(B)** An organismal phylogeny focused on γ- (green) and β-proteobacteria (purple), rooted with an ε-proteobacterium, *Campylobacter jejuni.* The Enterobacteriaceae are highlighted in green. Fully annotated version of this tree is presented in Supplementary Figure S4. **(C)** The flagellar phylogeny of the γ- (green) and β-proteobacteria (purple). Note the shift in position of the Enterobacteriaceae (highlighted in green) from the γ-proteobacterial clade to within the β-proteobacterial clade. Rooted with *Campylobacter jejuni.* Fully annotated version of this tree is presented in Supplementary Figure S5. **(D)** The *Bordetella bronchiseptica* flagellar gene cluster is arranged in one continuous genetic locus.

To understand the differences between the peritrichous flagella of the enteric γ-proteobacteria and the polar and lateral flagella of other γ-proteobacteria, we determined a flagellar phylogeny across > 90 species manually selected based on relevance and diversity by concatenating the protein sequences of their core flagellar proteins. For this we selected 12 core flagellar proteins, FlgI, FlgC, FliE, FliF, FlhA, FlhB, FliI, FliP, FliQ, FliR, FliG, and FliM (Figure 1A and Supplementary Figure S1), due to their ubiquity, ease of identification, core structural roles, absence of poorly characterized paralogous duplications, and absence of evidence of horizontal transfers. To assess the validity of our concatenated phylogeny, we examined our individual protein phylogenies. The phylogenies of 10 proteins, FlgI, FlgC, FliE, FliF, FlhA, FlhB, FliI, FliP, FliQ, and FliR, resembled the concatenated phylogeny (Supplementary Figure S2), as did the phylogenies of FliG and FliM except those from the γ-proteobacterial lateral motor, which were poorly resolved and with long branch lengths (Figure 1 and Supplementary Figure S3). Because FliG and FliM are components of the chemotaxis-associated C-ring, which has lost chemotactic ability in lateral motors, we speculate that the poorly resolved phylogeny of FliG and FliM in the lateral motor may be due to removal of functional constraints, leading to rapid sequence drift. Phylogenies calculated by omitting these proteins did not affect any of our subsequent conclusions.

We found that the polar and lateral γ-proteobacterial motors clustered together, but the peritrichous enteric motor instead branched from within the β-proteobacterial motors, suggesting horizontal acquisition from a member of the β-proteobacteria (Figure 1A), and we focused on a phylogeny of 48 β- and γ-proteobacteria for further inspection (Figures 1B,D and Supplementary Figures S4, S5). Reconstructing the motor phylogeny after removing FliG and FliM did not change this branching of the enteric motor from the β-proteobacterial motors. Our results suggest that the lateral and polar γ-proteobacterial systems diverged at a duplication event: the polar system sub-functionalized via structural elaboration and polar localization, while the non-chemotactic lateral system retained a structure resembling the common ancestral system of β/γ-proteobacteria but lost chemotactic ability.

The enteric motor branches from within a cluster of β-proteobacteria that belong to the *Burkholderiaceae* family (Parks et al., 2018), which includes *Bordetella, Cupriavidus* (ex-*Ralstonia*), and *Burkholderia*. Notably, flagellar genes in *Bordetella* species including *Bordetella bronchiseptica, B. avium*, and *B. parapertussis* are located at a single chromosomal locus (Figure 1D), suggesting a mechanism for the wholesale transfer of a single DNA fragment. This transfer would be difficult with other bacteria whose flagellar genes are fragmented across the genome. This putatively transferred chromosomal locus also includes the chemotaxis system that controls flagellar navigation. Furthermore, *cheD* is found outside the *che* locus in *Bordetella*; correspondingly, the enteric *che* systems also lack this component, presumably because only the physical *che* locus was transferred, and genes outside this locus—such as *cheD—*were not (Wuichet and Zhulin, 2010). Simultaneous transfer of the co-evolved, inter-dependent flagellar and sensory systems was likely more immediately useful to the recipient than transfer of individual systems alone. Synteny within operons has previously been shown to be strikingly similar between *Bordetella* and enteric flagella, although the significance of this was not reported (Liu and Ochman, 2007).

The γ-proteobacterial genus *Plesiomonas* phylogenetically branches from the base of the enterics yet retains native γ-proteobacterial and polar and lateral motors. Their presence in *Plesiomonas* suggests that an ancestral enteric γ-proteobacterium lost polar flagella and acquired the β-proteobacterial flagellum, although the order of these events cannot be inferred; some enterics retain lateral flagella (Ren et al., 2005), suggesting either selective loss, or reacquisition after species radiation. *Plesiomonas* is primarily aquatic yet also causes gastroenteritis, like many enterics, and whether *Plesiomonas* should be classified as an enteric (Enterobacteriaceae) remains controversial (Janda et al., 2016). The lack of diversity in the *Plesiomonas* genus relative to the other enterics could be explained by rapid diversification of enteric species facilitated by acquisition of the β-proteobacterial flagellum, although there may be other explanations.

**FIGURE 2:**
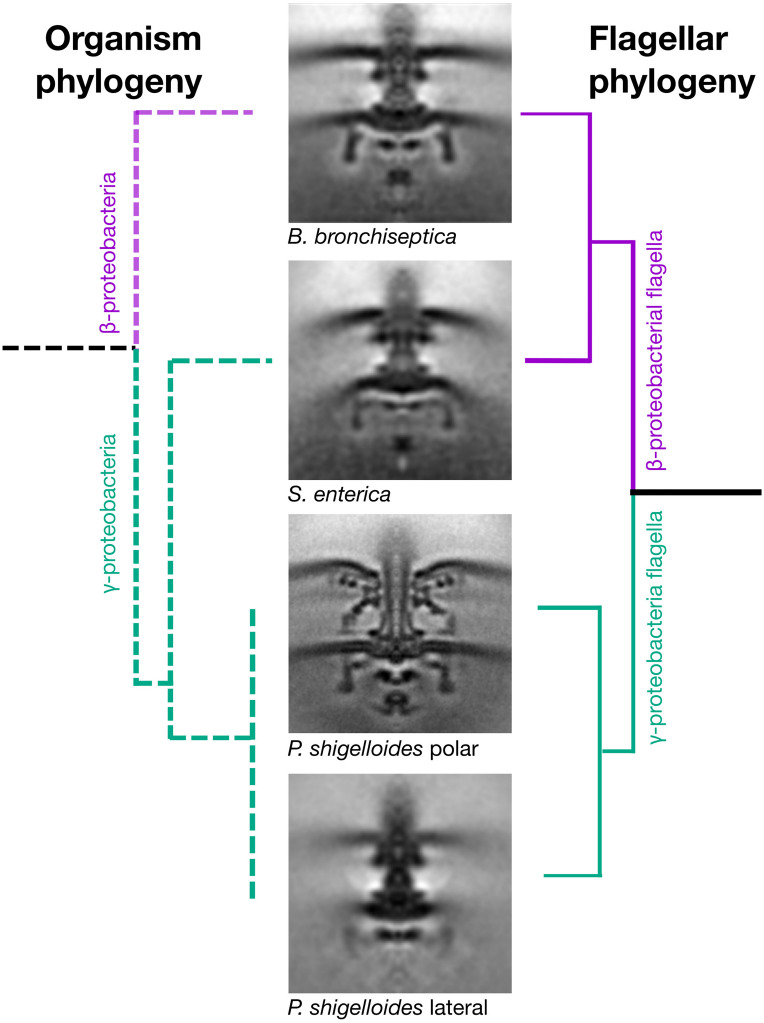
Horizontal transfer of flagellar genes is confirmed by cryo electron tomography and subtomogram averaging. Subtomogram averages of the “donor” (*Bordetella bronchiseptica*) and “acceptor” (*Plesiomonas shigelloides*) strains confirms that the γ-proteobacterium, *Salmonella enterica*, has a flagellar motor that is structurally similar to the β-proteobacterium, *B. bronchiseptica*, and structurally distinct from the two motors of its close relative, the γ-proteobacterium *P. shigelloides.* The organism phylogeny (left) and flagellar phylogeny (right) are inconsistent as the γ-proteobacterium, *Salmonella enterica*, has a β-proteobacterial motor. Middle: Subtomogram averages of *B. bronchiseptica* (EMD-4999), *S. enterica* (EMD-3154), *P. shigelloides* polar motor (EMD-10057) and *P. shigelloides* lateral motor (EMD-10000). Note the faint C-ring density in the lateral motor. Subtomogram average boxes are 100 × 100 nm. Unsymmetrized structures are shown in Supplementary Figure S7.

To understand the significance of this horizontal transfer, we sought to compare motor structures of *Bordetella*, *Salmonella*, and *Plesiomonas* using electron cryo-tomography and subtomogram averaging. We chose *Bordetella bronchiseptica* as a representative descendant of the ancestral β-proteobacterial donor, and used a Δ*bvgS* deletion to enable flagellation under lab conditions, as wildtype *B. bronchiseptica* uses its BvgAS two-component regulatory system to repress flagellar expression except under specific environmental conditions (Akerley et al., 1992). To reduce sample thickness, we gently deflated *B. bronchiseptica* cells with penicillin, and applied subtomogram averaging to 205 motors from 520 tomograms. Semblance of the *B. bronchiseptica* β-proteobacterial motor with the *Salmonella* and *E. coli* motors was consistent with a horizontal transfer (Figure 2), with comparable C-ring radii (20 nm), inter-membrane distances (29 nm), MS-ring and P-ring spacing (15 nm), and distance from the P-ring to the outer membrane (10 nm) (Rossmann and Beeby, 2018). As with the *Salmonella* motor, we could not discern stator complex densities in our subtomogram average structure, indicating that *B. bronchiseptica* stator complexes are dynamic.

We recently determined the structure of the polar motor from *P. shigelloides* (Ferreira et al., 2019) (Figure 2). This motor has a ring of 13 stator complexes, similar to the closely related *Vibrio fischeri* (Beeby et al., 2016) and *Vibrio alginolyticus* (Zhu et al., 2017), in contrast to the dynamic stator complexes of *Salmonella* and *B. bronchiseptica*, and likely explained by high stator complex occupancy. These polar motors have large disks required for stator complex assembly associated with their high torque and speed.

We next determined the structure of the lateral motor of *P. shigelloides*. We triggered lateral motor assembly by inhibiting polar motor function using Phenamil (Kawagishi et al., 1996), and thinned cells by penicillin deflation. To ensure identification of lateral motors we also deleted the polar flagellar filament Δ*fliC* and selected only motors with attached flagella; these motors were also distinguished by lateral placement, absence of periplasmic disks, and indistinct or absent C-rings. Subtomogram averaging of 317 motors from 632 tomograms revealed a well-resolved core, but absent stator complexes, and an indistinct C-ring (Figure 2). The indistinct C-ring is likely not a dissociation artifact from penicillin deflation, as laterally flagellated cells remained motile after deflation, polar flagella in the deflated cells had clearly resolved C-rings, *B. bronchiseptica* cells and previously imaged *E. coli* and *Salmonella* cells deflated using the same protocol had clearly resolved C-rings (Chen et al., 2011), and there was no correlation between lateral C-ring presence in tomograms and the extent of cell lysis. We conclude that lateral motor C-rings are more dynamic, and lower-occupancy, than C-rings from other motors.

The indistinct lateral motor C-ring is likely a result of the loss of chemotactic ability by lateral motors (Bubendorfer et al., 2014), consistent with the divergence of C-ring components found in our phylogenetic studies (Supplementary Figure S3). In most flagellar motors the C-ring plays dual roles as a switch complex, responding to phosphorylated CheY from chemoreceptors; and anchor for the FliHIJ complex (Chen et al., 2011; Abrusci et al., 2013). Because the lateral C-ring is only required to anchor FliHIJ, it may have evolved a more dynamic architecture than chemotactic motors, assembling as needed (Terashima et al., 2018). This is reminiscent of the non-rotating *Salmonella* SPI-1 injectisome “C-ring,” which also has the sole function of anchoring corresponding FliHIJ homologs (Hu et al., 2017). Consistent with this we did not resolve FliI in lateral motors, indicating that it, too, is present at lower occupancy.

We suggest that horizontal transfer of the β-proteobacterial flagellar motor to the enterics highlights a principle of evolution: having evolved, complexity is difficult to reverse. Duplication and sub-functionalization of the γ-proteobacterial motors yielded a polar system, which provided high torque and speed in aquatic habitats; and a lateral system, which provided surface swarming motility. This sub-functionalization and niche optimization came at the cost of the flexibility provided by the β-proteobacterial motor, as these specialized motors would be incapable of on-the-fly adjustment to the varied niches occupied by enteric bacteria. The only tractable evolutionary mechanism to provide the benefit of a generalist motor may have been to wholesale replace the specialized motors with a horizontally-transferred motor instead of re-generalization of the existing motors. This transfer was facilitated by co-occurrence of flagellar genes together in one region of DNA in an ancestral β-proteobacterium.

Indeed, we speculate that such a transfer may have accompanied the development of the enteric lifestyle of the enteric γ-proteobacteria. Acquisition of the β-proteobacterial motor provided a single, dynamic flagellum capable of adjusting to diverse environments and viscosities. Indeed, *Bordetella’s* role as a mucosal respiratory tract pathogen demonstrates the β-proteobacterial motor’s ability to colonize mucosal surfaces (Mattoo and Cherry, 2005; Solans and Locht, 2019). Dynamic stator complexes would facilitate optimal energy consumption, with stator complexes incorporating only as needed (Berg, 2017), at the cost of lower speeds and torques that may have been less important in an gastrointestinal tract than the higher flow rates of an aquatic environment. Because polar motors are usually sodium-driven, the proton-driven β-proteobacterial peritrichous motor could have benefited bacteria moving away from a high-salt aquatic niche.

While horizontal flagellar transfers have previously been described, no studies have recognized that the best-studied flagellar system, that of the enteric γ-proteobacteria, is a horizontal acquisition. An early comprehensive survey by Liu & Ochman noted the superficially similar transfer of a lateral (not to be confused with peritrichous) γ-proteobacteria flagellar system (there termed “secondary,” without highlighting that they are lateral systems from enteric γ-proteobacteria) to a β-proteobacterium, and also included a phylogenetic tree that depicted yet did not discuss our finding (Liu and Ochman, 2007). This oversight is also evident in a contemporaneous study describing the transfer of a polar flagellar system from a γ-proteobacterial donor to the α-proteobacterium *Rhodobacter sphaeroides* (Poggio et al., 2007), in which a figure depicting *E. coli* branching from a clade that included *Bordetella bronchiseptica* was not discussed. These and our results highlight the surprising complexity and frequency of wholesale transfers of flagellar systems, with the γ-proteobacteria duplicating their system to form polar (later transferred to an α-proteobacterium) and lateral (later transferred to a β-proteobacterium) systems, while a different β-proteobacterium transferred its (distinct) peritrichous system to the enteric clade of the γ-proteobacteria to supplant their native polar and lateral systems.

Our results show that the model flagellum is not native to its model organism host. Our finding resolves the long-standing paradox that different closely related γ-proteobacteria have different types of flagella. Furthermore, it shows a rare case by which specialization can be reversed by wholesale replacement with more generalized machinery. Our results may also help understand the evolution of the enterics, a family of diverse pathogens, and suggest development of novel, pathogen-specific drugs that target the β-proteobacterial-type motor.

## Materials and Methods

### Strains

**Table:** 

Bordetella bronchiseptica Δ*bvgS*	Andrew Preston, University of Bath

Plesiomonas shigelloides Δ*fliC*	Ferreira et al., 2019

### Bacterial Growth

Cells from fresh LB plates were grown overnight (36 h for *B. bronchiseptica*) in LB at 37°C, 290 RPM. Cells were diluted 1:100 into fresh LB and grown until OD_600_ ∼ 0.6. Prior to vitrification, cells were incubated with 466 IU/ml Penicillin G for half of one doubling time.

### Phylogenetic Analysis

Complete, annotated genomes (91 for Figure 1A or 48 for Figures 1B,C, species listed in Supplementary Table S1) were exported from GenBank (National Center for Biotechnology Information (NCBI)) and stored in WebApollo (Lee et al., 2013). Proteins were identified or annotations were confirmed by BLASTP (Altschul et al., 1990). Concatenated phylogenies were determined following our previously published protocols (Chaban et al., 2018). In brief, flagella (FlgI, FlgC, FliE, FliF, FlhA, FlhB, FliI, FliP, FliQ, and FliR) and ribosomal protein (RplA, RplB, RplC, RplD, RplE, RplF, RplI, RplJ, RplK, RplM, RplN, RplO, RplP, RplQ, RplR) sequences (see Supplementary Data Sheet S1) were aligned using Fast Statistical Alignment (FSA) (Bradley et al., 2009). The T-Coffee Suite was used to determine informative positions using the transitive consistence score (TCS) (Notredame et al., 2000; Di Tommaso et al., 2011). Sequences were concatenated in Seaview (Gouy et al., 2010). The Maximum-likelihood approach was used within Garli (Bazinet et al., 2014) using the Jones, Taylor and Thornton (JTT) amino acid substitution rates (Jones et al., 1994). Trees were created from the best of ten replicates. A topology improvement of 0.0001 in the lnL score and termination criterion of 100,000 were used. For the overall trees: 1,000 bootstraps were calculated with a topology improvement of 0.01 in the lnL score and termination criterion of 10,000. For the individual flagellar trees: 100 bootstraps were calculated with a topology improvement of 0.01 in the lnL score and termination criterion of 10,000. Bootstraps were added to the tree using SumTrees (SumTrees, 2019) and trees were visualized in FigTree (FigTree, 2017).

### Cryo-grid Preparation

Cell pellets were mixed with gold fiducials coated in BSA. 3μl of the cell/fiducial mixture was applied to freshly glow discharged R2/2, 300 mesh copper Quantifoil R2/2 grids (Quantifoil Micro Tools GmbH, Germany). A Vitrobot Mark IV was used to freeze grids in an ethane/propane cryogen at 100% humidity.

### Tilt-Series Collection

Tilt-series of *B. bronchiseptica* and *P. shigelloides* lateral motors were collected on an FEI F20 with a Falcon II detector (Thermo Fisher Scientific (formerly FEI), Hillsboro, OR, United States). A total fluence of 120 e-/Å^2^ was used and a defocus of between −3 and −4.5 μm. Tilt-series were collected over a tilt range of ± 53° with 3° tilt increments and a pixel size of 8.28 Å.

### Tomogram Reconstruction and Subtomogram Averaging

Tomograms of *B. bronchiseptica* and *P. shigelloides* lateral motors were reconstructed automatically using RAPTOR (Amat et al., 2008) and IMOD (Kremer et al., 1996).

Subtomograms were picked manually (205 subtomograms picked from 520 tomograms for *B. bronchiseptica* and 317 subtomograms picked from 632 tomograms for *P. shigelloides*). Template-free alignment was carried out in PEET by superimposing all manually picked subtomograms allowing no shifts or rotations for an initial reference. As no symmetry was observed, and to better visualize the overall motor profile, 100-fold rotational averaging was applied using custom scripts.

## Data Availability Statement

The datasets presented in this study can be found in online repositories. The names of the repository/repositories and accession number(s) can be found below: https://www.ebi.ac.uk/pdbe/emdb/, EMD-10000; https://www.ebi.ac.uk/pdbe/emdb/, EMD-4999.

## Author Contributions

JF, IC, BQ, KW, and MB designed experiments. JF, IC, MA, TZ, and BQ conducted experiments. JF, KW, and MB wrote the manuscript. All authors contributed to the article and approved the submitted version.

## Conflict of Interest

The authors declare that the research was conducted in the absence of any commercial or financial relationships that could be construed as a potential conflict of interest.

## Supplementary Material

Supplementary Figure 1 Fully annotated wide-scale flagellar phylogeny tree from Figure 1A. An unrooted, wide-scale tree of flagellar phylogeny. The Enterobacteriaceae (*Salmonella enterica* and *Escherichia coli)* are not clustered with the other γ-proteobacteria (green), but are clustered within the β-proteobacteria (purple).

Supplementary Figure 2 Unrooted trees of individual flagellar proteins. Individual flagellar proteins used in the concatenated flagellar tree have similar topologies with each other and with the concatenated tree. β-proteobacteria in purple, γ-proteobacteria in green, lateral flagella highlighted in pink. 1 = polar, 2 = lateral.

Supplementary Figure 3 Trees made using the flagellar proteins C-ring proteins. Unrooted FliG and FliM trees differ from other flagellar protein trees (depicted in Supplementary Figure S2) or the concatenated tree (Figure 1C) due to long branches in the lateral flagellar clade (highlighted in pink). 1 = polar, 2 = lateral.

Supplementary Figure 4 Fully annotated organismal phylogeny tree from Figure 1B. β-proteobacteria in purple, γ-proteobacteria in green, Enterobacteriaceae highlighted in green. Rooted with the ε-proteobacterium, *Campylobacter jejuni*. Bootstrap values indicated on nodes.

Supplementary Figure 5 Fully annotated flagellar phylogeny tree from Figure 1C. β-proteobacteria in purple, γ-proteobacteria in green, Enterobacteriaceae highlighted in green. Rooted with the ε-proteobacterium, *Campylobacter jejuni*. Bootstrap values indicated on nodes.

Supplementary Figure 6 Example tomograms. **(A)** Slice through a representative tomogram of *B. bronchiseptica* with a peritrichous motor pointed out in green. **(B)** Slice through a representative tomogram of *P. shigelloides* with the lateral motor pointed out in purple and the polar motors with only hooks (filament protein, FliC, is deleted) in yellow.

Supplementary Figure 7 Subtomogram averages prior to C100 rotational averaging. **(A)** Slice through *Bordetella bronchiseptica* motor unsymmetrised subtomogram average, **(B)** Slice through *Plesiomonas shigelloides* lateral motor unsymmetrised subtomogram average. Boxes are 100 nm × 100 nm.

## References

[B1] Reid S. W. Leake M. C. Chandler J. H. Lo C.-J. Armitage J. P. Berry R. M. (2006). The maximum number of torque-generating units in the flagellar motor of *Escherichia coli* is at least 11. *Proc. Natl. Acad. Sci. U S A.* 103 8066–8071. 10.1073/pnas.0509932103 16698936 PMC1472430

[B2] Tipping M. J. Delalez N. J. Lim R. Berry R. M. Armitage J. P. (2013). Load-dependent assembly of the bacterial flagellar motor. *mBio* 4 1–6. 10.1128/mBio.00551-13 23963182 PMC3747592

[B3] Lee S. Y. Cho H. S. Pelton J. G. Yan D. Henderson R. K. King D. S. (2001). Crystal structure of an activated response regulator bound to its target. *Nat. Struct. Biol.* 8 52–56. 10.1038/83053 11135671

[B4] Berg H. C. Brown D. A. (1972). Chemotaxis in *Escherichia coli* analysed by Three-dimensional Tracking. *Nature* 239 500–504. 10.1038/239500a0 4563019

[B5] Silverman M. Simon M. (1974). Flagellar rotation and the mechanism of bacterial motility. *Nature* 249 73–74. 10.1038/249073a0 4598030

[B6] Beeby M. Ribardo D. A. Brennan C. A. Ruby E. G. Jensen G. J. Hendrixson D. R. (2016). Diverse high-torque bacterial flagellar motors assemble wider stator rings using a conserved protein scaffold. *Proc. Natl. Acad. Sci.* 2016:201518952. 10.1073/pnas.1518952113 26976588 PMC4822576

[B7] Zhu S. Nishikino T. Hu B. Kojima S. Homma M. Liu J. (2017). Molecular architecture of the sheathed polar flagellum in Vibrio alginolyticus. *Proc. Natl. Acad. Sci. U S A.* 114 10966–10971. 10.1073/pnas.1712489114 28973904 PMC5642721

[B8] Terashima H. Koike M. Kojima S. Homma M. (2010). The Flagellar Basal Body-Associated Protein FlgT Is Essential for a Novel Ring Structure in the Sodium-Driven Vibrio Motor. *J. Bacteriol.* 192 5609–5615. 10.1128/JB.00720-10 20729351 PMC2953672

[B9] Terashima H. Fukuoka H. Yakushi T. Kojima S. Homma M. (2006). The Vibrio motor proteins, MotX and MotY, are associated with the basal body of Na-driven flagella and required for stator formation. *Mol. Microbiol.* 62 1170–1180. 10.1111/j.1365-2958.2006.05435.x 17038120

[B10] Bubendorfer S. Koltai M. Rossmann F. Sourjik V. Thormann K. M. (2014). Secondary bacterial flagellar system improves bacterial spreading by increasing the directional persistence of swimming. *Proc. Natl. Acad. Sci.* 111 11485–11490. 10.1073/pnas.1405820111 25049414 PMC4128160

[B11] Xie L. Altindal T. Chattopadhyay S. Wu X.-L. (2011). Bacterial flagellum as a propeller and as a rudder for efficient chemotaxis. *Proc. Natl. Acad. Sci.* 108 2246–2251. 10.1073/pnas.1011953108 21205908 PMC3038696

[B12] Fujii M. Shibata S. Aizawa S.-I. (2008). Polar, Peritrichous, and Lateral Flagella Belong to Three Distinguishable Flagellar Families. *J. Mol. Biol.* 379 273–283. 10.1016/j.jmb.2008.04.012 18455187

[B13] Parks D. H. Chuvochina M. Waite D. W. Rinke C. Skarshewski A. Chaumeil P.-A. (2018). A standardized bacterial taxonomy based on genome phylogeny substantially revises the tree of life. *Nat. Biotechnol.* 36 996–1004. 10.1038/nbt.4229 30148503

[B14] Wuichet K. Zhulin I. B. (2010). Origins and diversification of a complex signal transduction system in prokaryotes. *Sci. Signal.* 3:ra50. 10.1126/scisignal.2000724 20587806 PMC3401578

[B15] Liu R. Ochman H. (2007). Origins of flagellar gene: Operons and secondary flagellar systems. *J. Bacteriol.* 189 7098–7104. 10.1128/JB.00643-07 17644605 PMC2045201

[B16] Ren C.-P. Beatson S. A. Parkhill J. Pallen M. J. (2005). The Flag-2 locus, an ancestral gene cluster, is potentially associated with a novel flagellar system from *Escherichia coli*. *J. Bacteriol.* 187 1430–1440. 10.1128/JB.187.4.1430-1440.2005 15687208 PMC545627

[B17] Janda J. M. Abbott S. L. McIver C. J. (2016). Plesiomonas shigelloides Revisited. *Clin. Microbiol. Rev.* 29 349–374. 10.1128/CMR.00103-15 26960939 PMC4786884

[B18] Akerley B. J. Monack D. M. Falkow S. Miller J. F. (1992). The bvgAS locus negatively controls motility and synthesis of flagella in Bordetella bronchiseptica. *J. Bacteriol.* 174 980–990. 1370665 10.1128/jb.174.3.980-990.1992 PMC206178

[B19] Rossmann F. M. Beeby M. (2018). Insights into the evolution of bacterial flagellar motors from high-throughput in situ electron cryotomography and subtomogram averaging. *Acta Crystallogr. Sect. Struct. Biol.* 74 585–594. 10.1107/S2059798318007945 29872008 PMC6096493

[B20] Ferreira J. L. Gao F. Z. Rossmann F. M. Nans A. Brenzinger S. Hosseini R. (2019). γ-*proteobacteria* eject their polar flagella under nutrient depletion, retaining flagellar motor relic structures. *PLoS Biol.* 17:e3000165. 10.1371/journal.pbio.3000165 30889173 PMC6424402

[B21] Kawagishi I. Imagawa M. Imae Y. McCarter L. Homma M. (1996). The sodium-driven polar flagellar motor of marine Vibrio as the mechanosensor that regulates lateral flagellar expression. *Mol. Microbiol.* 20 693–699. 8793868 10.1111/j.1365-2958.1996.tb02509.x

[B22] Chen S. Beeby M. Murphy G. E. Leadbetter J. R. Hendrixson D. R. Briegel A. (2011). Structural diversity of bacterial flagellar motors. *EMBO J.* 30 2972–2981. 10.1038/emboj.2011.186 21673657 PMC3160247

[B23] Abrusci P. Vergara–Irigaray M. Johnson S. Beeby M. D. Hendrixson D. Roversi P. (2013). Architecture of the major component of the type III secretion system export apparatus. *Nat. Struct. Mol. Biol.* 20 99–104. 10.1038/nsmb.2452 23222644 PMC3537844

[B24] Terashima H. Kawamoto A. Tatsumi C. Namba K. Minamino T. Imada K. (2018). In Vitro Reconstitution of Functional Type III Protein Export and Insights into Flagellar Assembly. *mBio* 9:18. 10.1128/mBio.00988-18 29946050 PMC6020293

[B25] Hu B. Lara-Tejero M. Kong Q. Galán J. E. Liu J. (2017). In Situ Molecular Architecture of the *Salmonella* Type III Secretion Machine. *Cell* 168 1065.e–1074.e. 10.1016/j.cell.2017.02.022 28283062 PMC5393631

[B26] Mattoo S. Cherry J. D. (2005). Molecular Pathogenesis, Epidemiology, and Clinical Manifestations of Respiratory Infections Due to Bordetella pertussis and Other Bordetella Subspecies. *Clin. Microbiol. Rev.* 18 326–382. 10.1128/CMR.18.2.326-382.2005 15831828 PMC1082800

[B27] Solans L. Locht C. (2019). The Role of Mucosal Immunity in Pertussis. *Front. Immunol.* 9:03068. 10.3389/fimmu.2018.03068 30692990 PMC6339907

[B28] Berg H. C. (2017). The flagellar motor adapts, optimizing bacterial behavior. *Protein Sci. Publ. Protein Soc.* 26 1249–1251. 10.1002/pro.3055 27679984 PMC5477541

[B29] Poggio S. Abreu-Goodger C. Fabela S. Osorio A. Dreyfus G. Vinuesa P. (2007). A Complete Set of Flagellar Genes Acquired by Horizontal Transfer Coexists with the Endogenous Flagellar System in Rhodobacter Sphaeroides. *J. Bacteriol.* 189 3208–3216. 10.1128/JB.01681-06 17293429 PMC1855832

[B30] Lee E. Helt G. A. Reese J. T. Munoz-Torres M. C. Childers C. P. Buels R. M. (2013). Web Apollo: a web-based genomic annotation editing platform. *Genome Biol.* 14:R93. 10.1186/gb-2013-14-8-r93 24000942 PMC4053811

[B31] Altschul S. F. Gish W. Miller W. Myers E. W. Lipman D. J. (1990). Basic local alignment search tool. *J. Mol. Biol.* 215 403–410. 10.1016/S0022-2836(05)80360-2 2231712

[B32] Chaban B. Coleman I. Beeby M. (2018). Evolution of higher torque in Campylobacter- type bacterial flagellar motors. *Sci. Rep.* 8:97. 10.1038/s41598-017-18115-1 29311627 PMC5758724

[B33] Bradley R. K. Roberts A. Smoot M. Juvekar S. Do J. Dewey C. (2009). Fast Statistical Alignment. *PLoS Comput Biol.* 5:e1000392. 10.1371/journal.pcbi.1000392 19478997 PMC2684580

[B34] Notredame C. Higgins D. G. Heringa J. T. - (2000). Coffee: A novel method for fast and accurate multiple sequence alignment. *J. Mol. Biol.* 302 205–217. 10.1006/jmbi.2000.4042 10964570

[B35] Di Tommaso P. Moretti S. Xenarios I. Orobitg M. Montanyola A. Chang J.-M. (2011). T-Coffee: a web server for the multiple sequence alignment of protein and RNA sequences using structural information and homology extension. *Nucleic Acids Res.* 39 W13–W17. 10.1093/nar/gkr245 21558174 PMC3125728

[B36] Gouy M. Guindon S. Gascuel O. (2010). SeaView version 4: A multiplatform graphical user interface for sequence alignment and phylogenetic tree building. *Mol. Biol. Evol.* 27 221–224. 10.1093/molbev/msp259 19854763

[B37] Bazinet A. L. Zwickl D. J. Cummings M. P. A. (2014). gateway for phylogenetic analysis powered by grid computing featuring GARLI 2.0. *Syst. Biol.* 63 812–818. 10.1093/sysbio/syu031 24789072 PMC4141202

[B38] Jones D. T. Taylor W. R. Thornton J. M. A. (1994). Model Recognition Approach to the Prediction of All-Helical Membrane Protein Structure and Topology. *Biochemistry* 33 3038–3049. 10.1021/bi00176a037 8130217

[B39] SumTrees (2019). *Phylogenetic Tree Summarization and Annotation — DendroPy 4.4.0 documentation.*. Available online at: https://dendropy.org/programs/sumtrees.html [accessed date 3, April 2019]

[B40] FigTree (2017). Available online at: http://tree.bio.ed.ac.uk/software/figtree/ (accessed date 2, April 2017).

[B41] Amat F. Moussavi F. Comolli L. R. Elidan G. Downing K. H. Horowitz M. (2008). Markov random field based automatic image alignment for electron tomography. *J. Struct. Biol.* 161 260–275. 10.1016/j.jsb.2007.07.007 17855124

[B42] Kremer J. R. Mastronarde D. N. McIntosh J. R. (1996). Computer visualization of three-dimensional image data using IMOD. *J. Struct. Biol.* 116 71–76. 10.1006/jsbi.1996.0013 8742726

