# Corrigendum: The “Jack-of-all-Trades” Flagellum From *Salmonella* and *E. coli* Was Horizontally Acquired From an Ancestral β-Proteobacterium

**DOI:** 10.3389/fmicb.2021.773675

**Published:** 2021-10-06

**Authors:** Josie L. Ferreira, Izaak Coleman, Max L. Addison, Tobias Zachs, Bonnie L. Quigley, Kristin Wuichet, Morgan Beeby

**Affiliations:** ^1^Department of Life Sciences, Imperial College London, London, United Kingdom; ^2^Department of Biomedical Informatics, Vanderbilt University Medical Center, Nashville, TN, United States

**Keywords:** bacterial flagella, electron cryotomography, molecular evolution, subtomogram averaging, horizontal gene transfer

In the original article, there was a mistake in Figure 1 as published. **We inadvertently uploaded an outdated version of this figure**. The corrected Figure 1 appears below.

**Figure 1:**
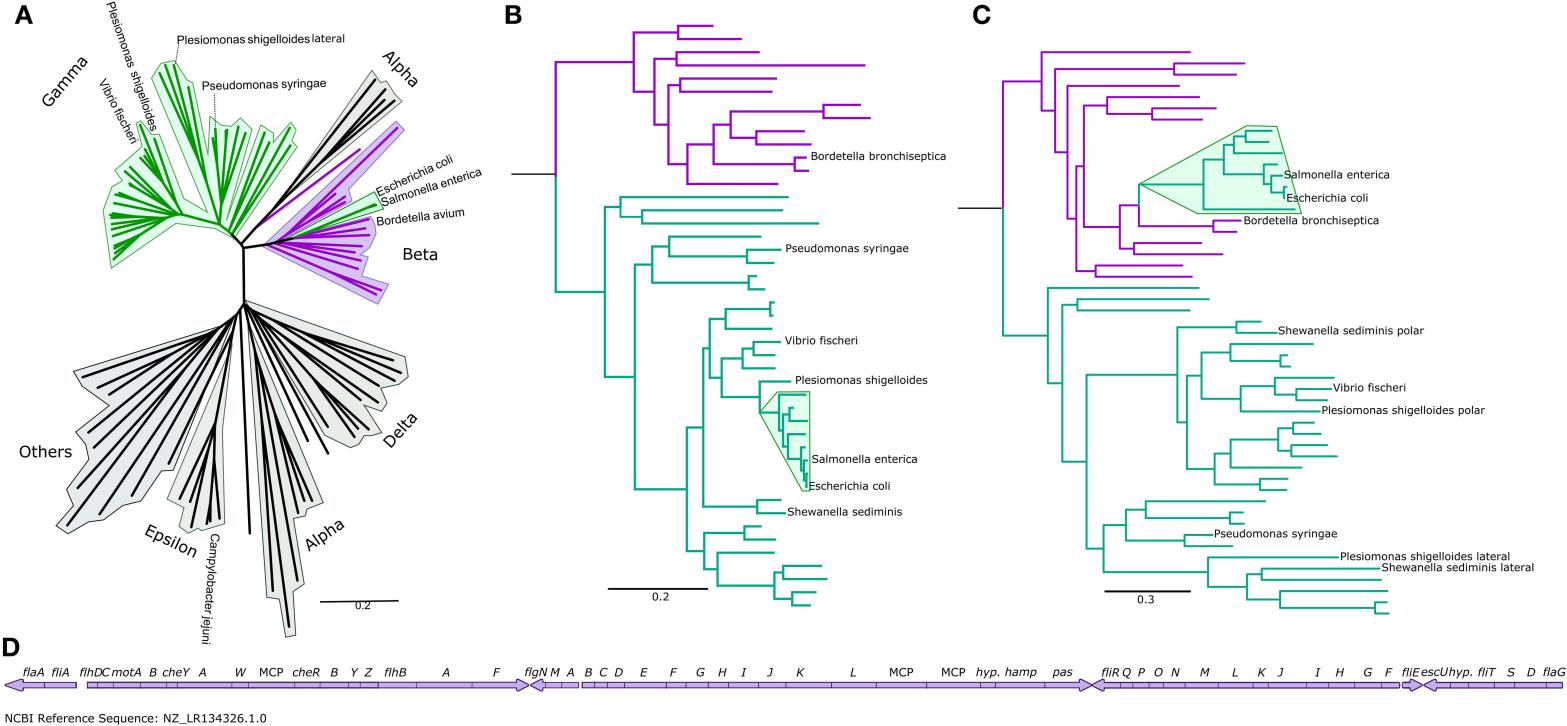
The Enterobacteriaceae have β-like motors. **(A)** An unrooted global flagellar phylogeny. γ-proteobacteria are highlighted in green: the enteric Enterobacteriaceae γ-proteobacteria (*Salmonella enterica* and *Escherichia coli*) are not clustered with the other γ-proteobacteria, but are clustered within the β-proteobacteria (purple). Fully annotated version of this tree is presented in Supplementary Figure S1. **(B)** An organismal phylogeny focused on γ- (green) and β-proteobacteria (purple), rooted with an ε-proteobacterium, *Campylobacter jejuni*. The Enterobacteriaceae are highlighted in green. Fully annotated version of this tree is presented in Supplementary Figure S4. **(C)** The flagellar phylogeny of the γ- (green) and β-proteobacteria (purple). Note the shift in position of the Enterobacteriaceae (highlighted in green) from the γ-proteobacterial clade to within the β-proteobacterial clade. Rooted with *Campylobacter jejuni*. Fully annotated version of this tree is presented in Supplementary Figure S5. **(D)** The *Bordetella bronchiseptica* flagellar gene cluster is arranged in one continuous genetic locus.

The authors apologize for this error and state that this does not change the scientific conclusions of the article in any way. The original article has been updated.

## Publisher's Note

All claims expressed in this article are solely those of the authors and do not necessarily represent those of their affiliated organizations, or those of the publisher, the editors and the reviewers. Any product that may be evaluated in this article, or claim that may be made by its manufacturer, is not guaranteed or endorsed by the publisher.

