# Supplementary figures and images for: The “Jack-of-all-Trades” Flagellum From Salmonella and E. coli Was Horizontally Acquired From an Ancestral β-Proteobacterium
Source: Front Microbiol. 2021 Mar 30;12:643180. doi: 10.3389/fmicb.2021.643180 (PMC8042155; doi:10.3389/fmicb.2021.643180)

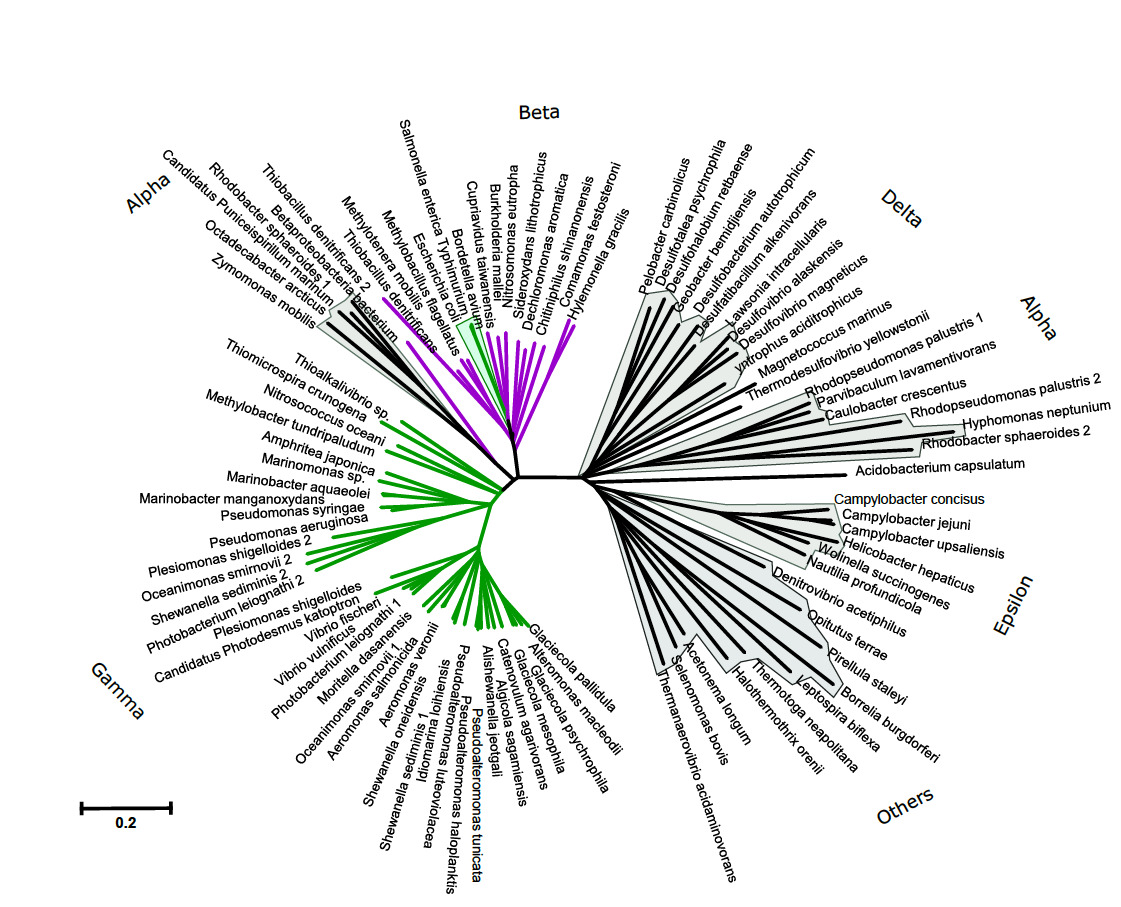

Supplement: Supplementary Figure 1 — Fully annotated wide-scale flagellar phylogeny tree from Figure 1A. An unrooted, wide-scale tree of flagellar phylogeny. The Enterobacteriaceae (Salmonella enterica and Escherichia coli) are not clustered with the other γ-proteobacteria (green), but are clustered within the β-proteobacteria (purple). [file Image_1.JPEG]

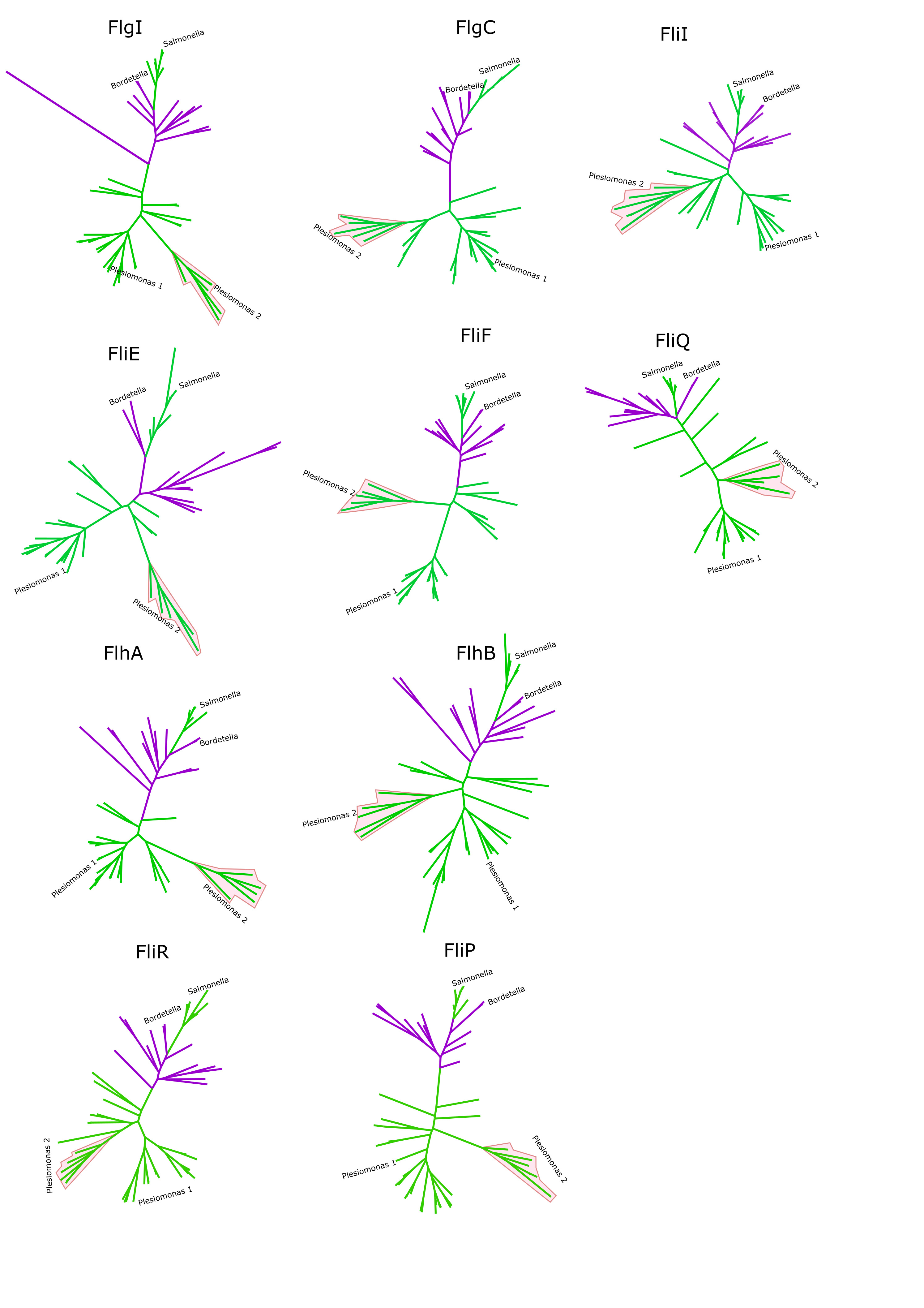

Supplement: Supplementary Figure 2 — Unrooted trees of individual flagellar proteins. Individual flagellar proteins used in the concatenated flagellar tree have similar topologies with each other and with the concatenated tree. β-proteobacteria in purple, γ-proteobacteria in green, lateral flagella highlighted in pink. 1 = polar, 2 = lateral. [file Image_2.JPEG]

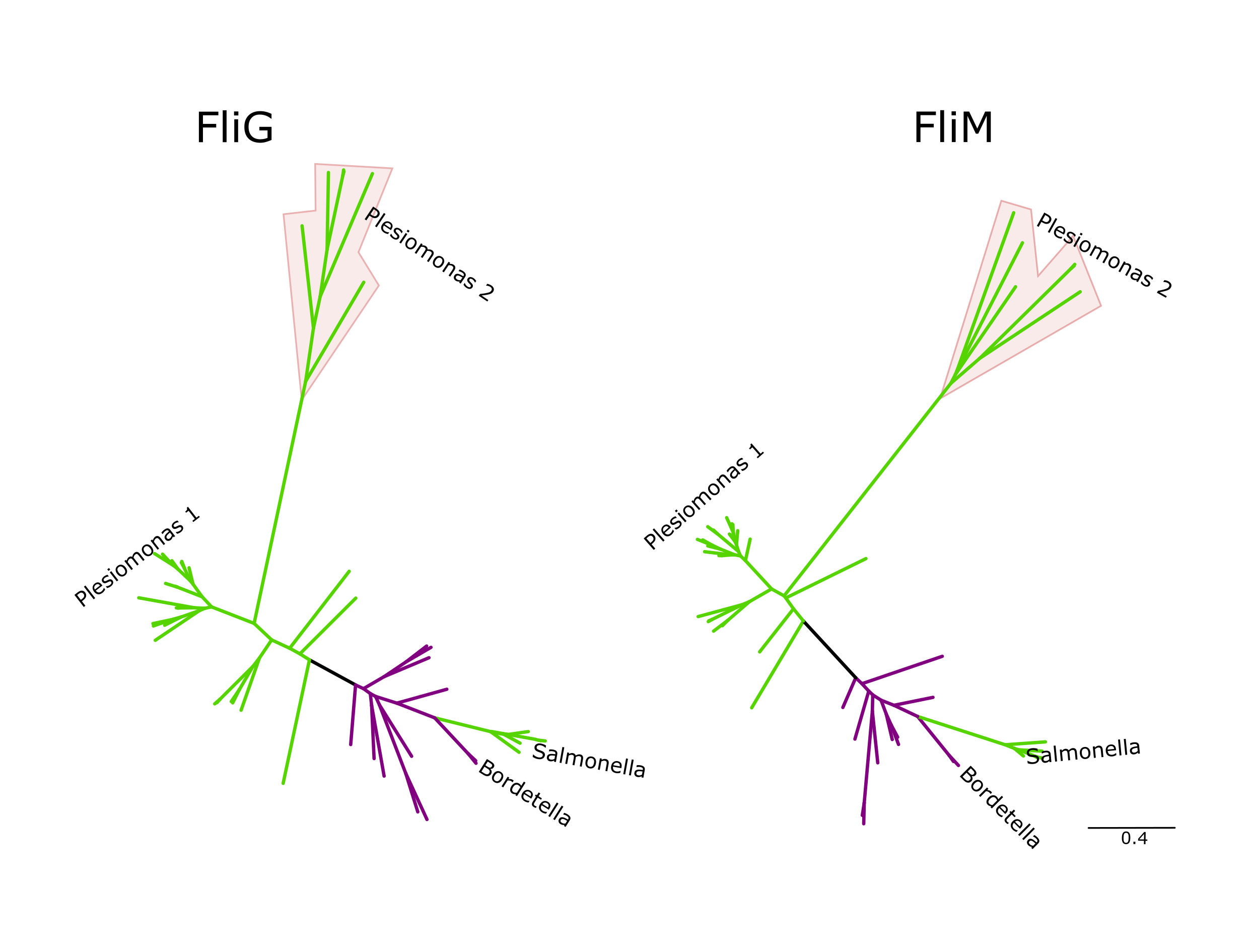

Supplement: Supplementary Figure 3 — Trees made using the flagellar proteins C-ring proteins. Unrooted FliG and FliM trees differ from other flagellar protein trees (depicted in Supplementary Figure S2) or the concatenated tree (Figure 1C) due to long branches in the lateral flagellar clade (highlighted in pink). 1 = polar, 2 = lateral. [file Image_3.JPEG]

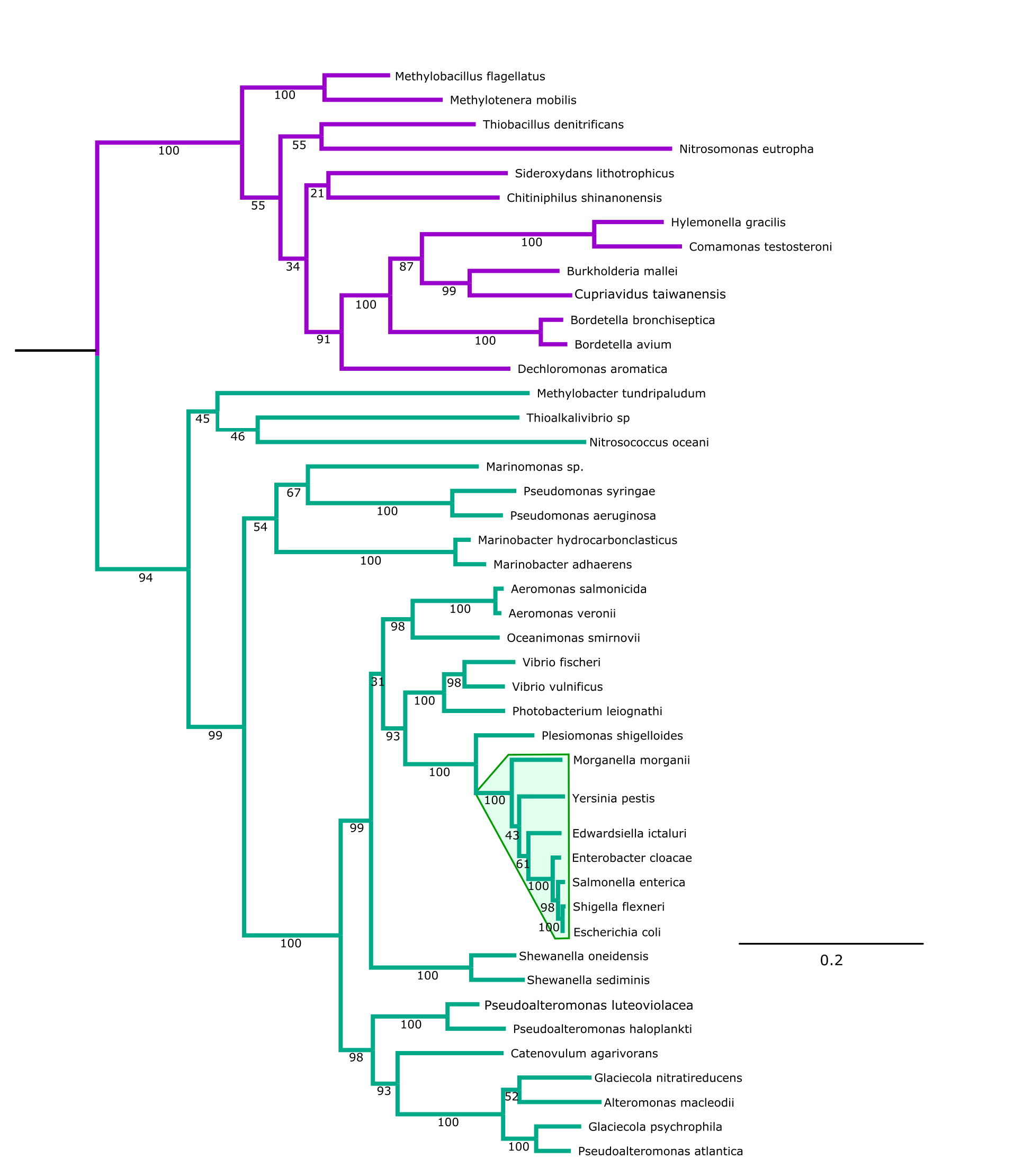

Supplement: Supplementary Figure 4 — Fully annotated organismal phylogeny tree from Figure 1B. β-proteobacteria in purple, γ-proteobacteria in green, Enterobacteriaceae highlighted in green. Rooted with the ε-proteobacterium, Campylobacter jejuni. Bootstrap values indicated on nodes. [file Image_4.JPEG]

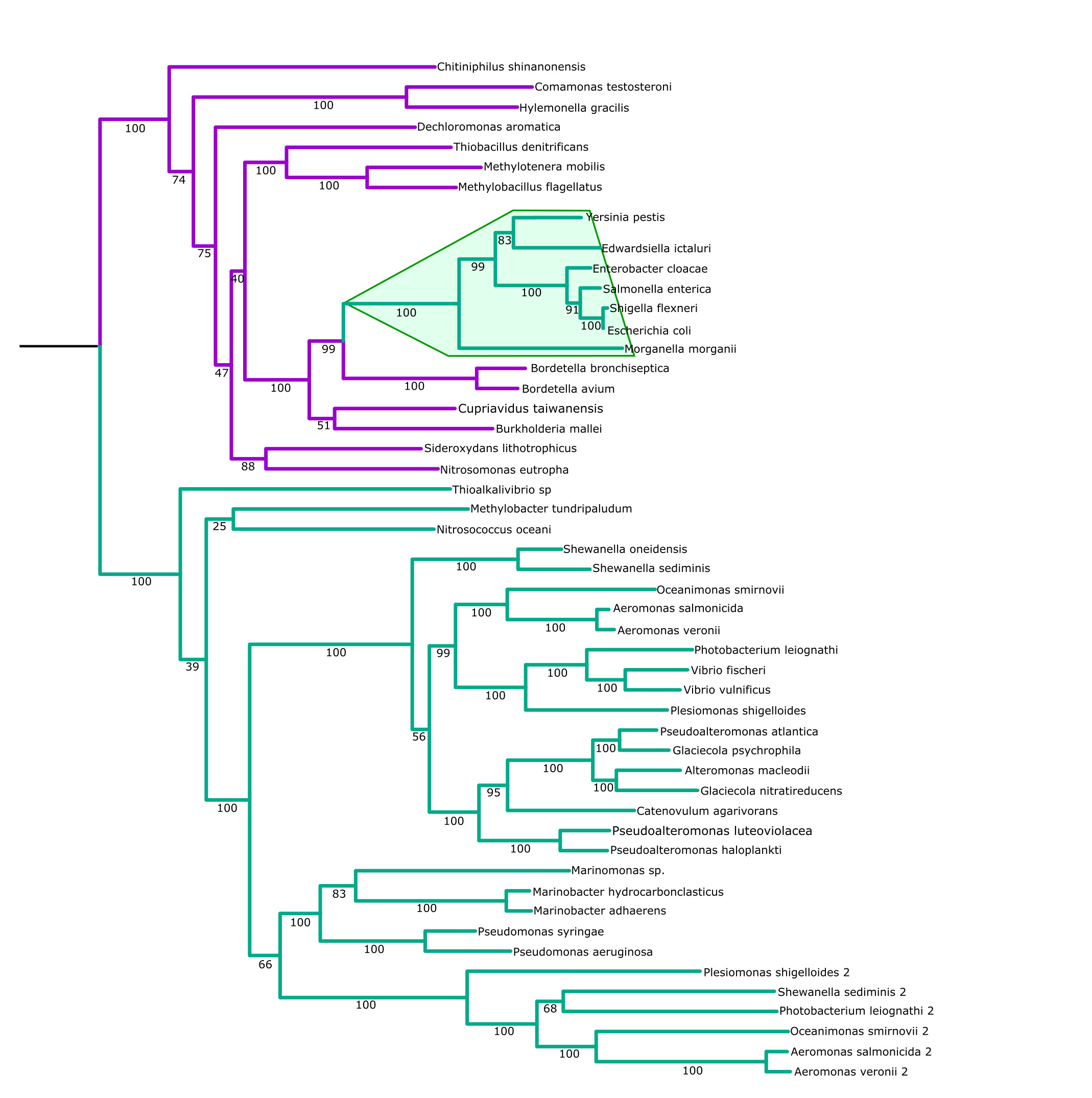

Supplement: Supplementary Figure 5 — Fully annotated flagellar phylogeny tree from Figure 1C. β-proteobacteria in purple, γ-proteobacteria in green, Enterobacteriaceae highlighted in green. Rooted with the ε-proteobacterium, Campylobacter jejuni. Bootstrap values indicated on nodes. [file Image_5.JPEG]

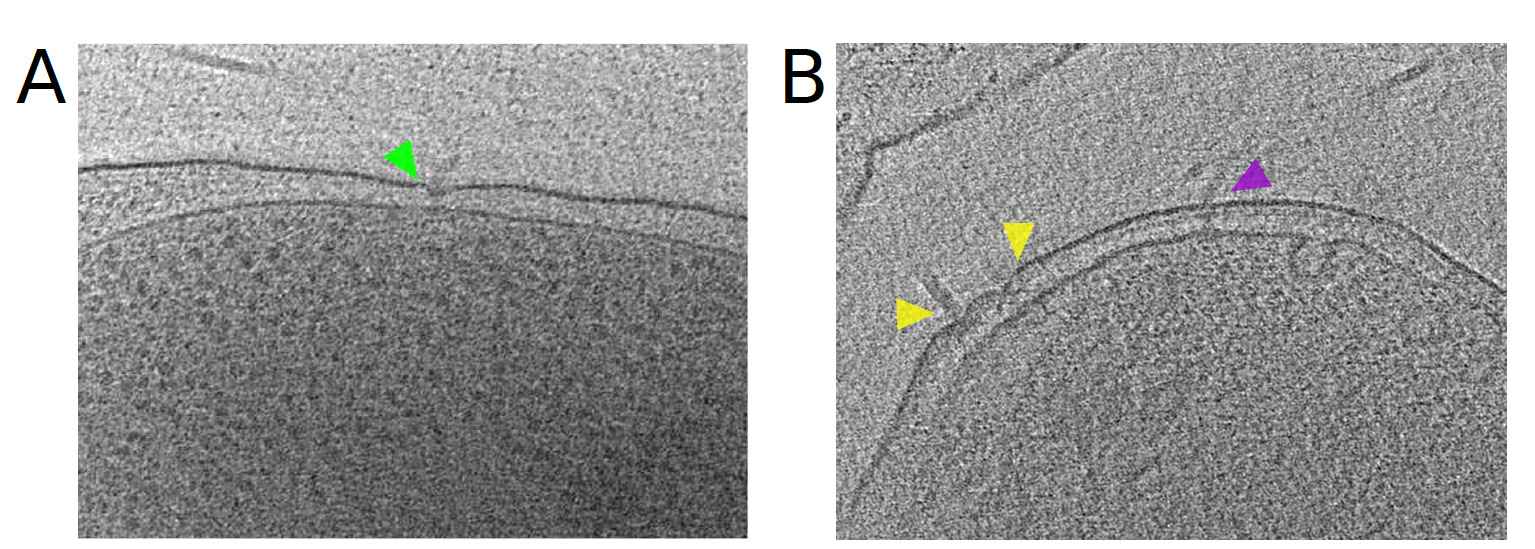

Supplement: Supplementary Figure 6 — Example tomograms. (A) Slice through a representative tomogram of B. bronchiseptica with a peritrichous motor pointed out in green. (B) Slice through a representative tomogram of P. shigelloides with the lateral motor pointed out in purple and the polar motors with only hooks (filament protein, FliC, is deleted) in yellow. [file Image_6.JPEG]

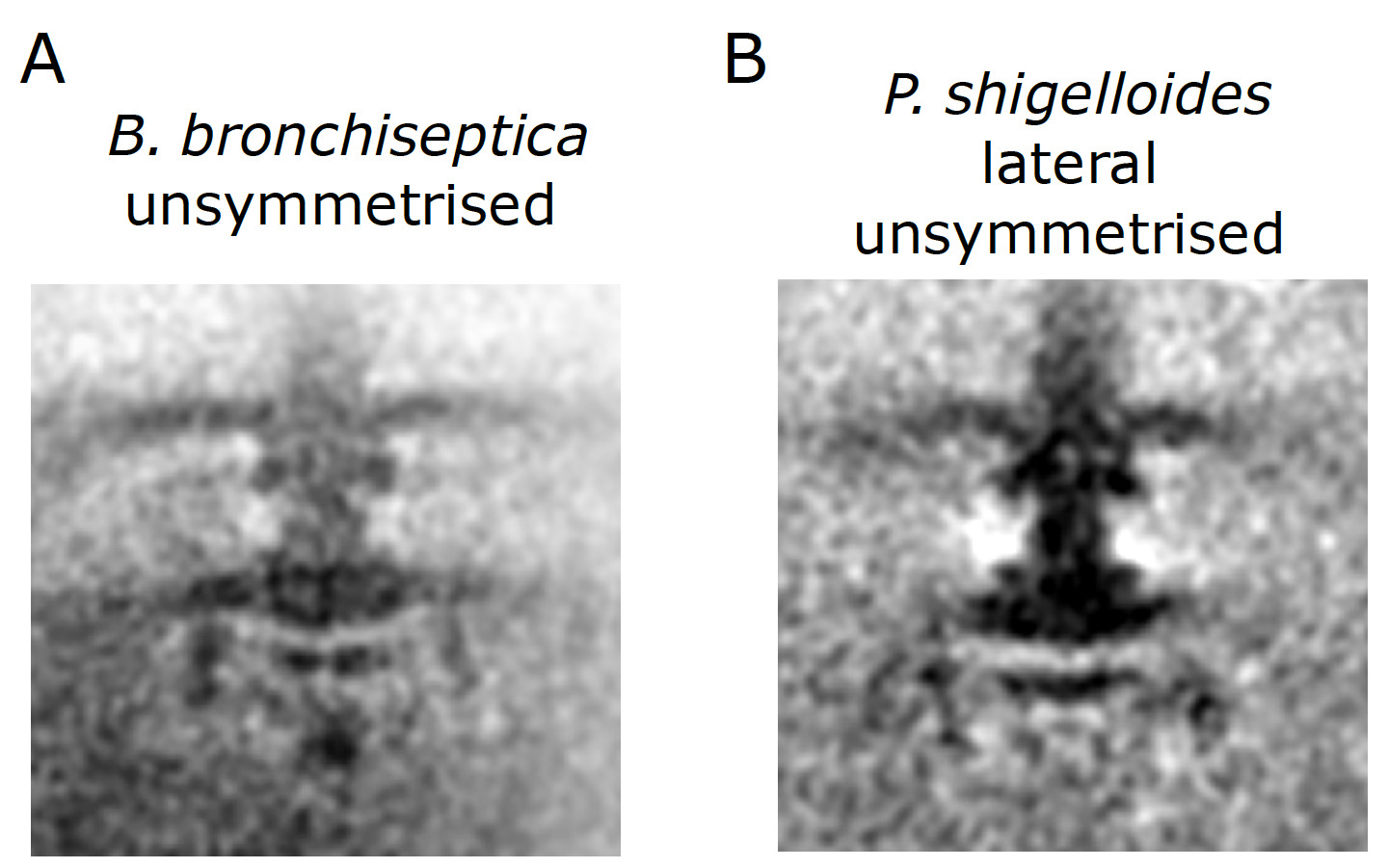

Supplement: Supplementary Figure 7 — Subtomogram averages prior to C100 rotational averaging. (A) Slice through Bordetella bronchiseptica motor unsymmetrised subtomogram average, (B) Slice through Plesiomonas shigelloides lateral motor unsymmetrised subtomogram average. Boxes are 100 nm × 100 nm. [file Image_7.JPEG]
